# The Reliability and Validity of the Clinical Perfectionism Questionnaire in Eating Disorder and Community Samples

**DOI:** 10.1017/S1352465814000629

**Published:** 2015-03-03

**Authors:** Sarah J. Egan, Roz Shafran, Michelle Lee, Christopher G. Fairburn, Zafra Cooper, Helen A. Doll, Robert L. Palmer, Hunna J. Watson

**Affiliations:** Curtin University, Australia; UCL Institute of Child Health, London, UK; University of Reading, UK; Oxford University, UK; Leicestershire Partnership NHS Trust and University of Leicester, UK; Curtin University, Australia, Child and Adolescent Health Service, Perth, Australia, University of North Carolina at Chapel Hill, USA, and The University of Western Australia, Australia

**Keywords:** Perfectionism, reliability, transdiagnostic, eating disorder, validity

## Abstract

**Background:** Clinical perfectionism is a risk and maintaining factor for anxiety disorders, depression and eating disorders. **Aims:** The aim was to examine the psychometric properties of the 12-item Clinical Perfectionism Questionnaire (CPQ). **Method:** The research involved two samples. Study 1 comprised a nonclinical sample (*n* = 206) recruited via the internet. Study 2 comprised individuals in treatment for an eating disorder (*n* = 129) and a community sample (*n* = 80). **Results:** Study 1 factor analysis results indicated a two-factor structure. The CPQ had strong correlations with measures of perfectionism and psychopathology, acceptable internal consistency, and discriminative and incremental validity. The results of Study 2 suggested the same two-factor structure, acceptable internal consistency, and construct validity, with the CPQ discriminating between the eating disorder and control groups. Readability was assessed as a US grade 4 reading level (student age range 9–10 years). **Conclusions:** The findings provide evidence for the reliability and validity of the CPQ in a clinical eating disorder and two separate community samples. Although further research is required the CPQ has promising evidence as a reliable and valid measure of clinical perfectionism.

## Introduction

Perfectionism has predominately been viewed as multidimensional and measured with the Frost Multidimensional Perfectionism Scale (FMPS; Frost, Marten, Lahart and Rosenblate, 1990) and the Hewitt and Flett Multidimensional Perfectionism Scale (HMPS; Hewitt and Flett, 1991). The FMPS consists of Personal Standards (PS), Concern over Mistakes (CM), Doubts about Actions (DA), Parental Expectations (PE), Parental Criticism (PC), and Organization (O). The HMPS consists of self-oriented perfectionism (SOP), other-oriented perfectionism (OOP) and socially-prescribed perfectionism (SPP). Studies have found a consistent two-factor solution; Evaluative Concerns (EC = CM, DA, PC, PE, SPP) and Positive Striving (PS = PS, O, SOP, OOP) (e.g. Bieling, Israeli and Antony, 2004).

Clinical perfectionism is considered to make a unique contribution to the perfectionism construct in addition to PS and EC, and is defined as striving to meet demanding standards despite negative consequences, and basing self-esteem on achievement (Shafran, Cooper and Fairburn, 2002). It is distinct because of the emphasis of clinical perfectionism as being self-worth dependent on striving and achievement at the core. There has been some contention over clinical perfectionism, particularly around the notion that it is unidimensional (e.g. Dunkley, Blankstein, Masheb and Grilo, 2006). However Egan, Wade and Shafran (2011) pointed out that Shafran et al. (2002) were not stating that striving for excellence in itself is negative, but that it is self-worth being dependent on striving and achievement of personally demanding standards that is important. Clinical perfectionism is a maintaining factor in the transdiagnostic model of eating disorders (Fairburn, Cooper and Shafran, 2003a). Furthermore, it is a transdiagnostic “construct” and is a risk and maintaining factor in eating disorders, anxiety disorders and depression (Egan et al., 2011). Treatment with cognitive behaviour therapy (CBT) (e.g. Shafran, Egan and Wade, 2010; Egan, Wade, Shafran and Antony, 2014) results in reductions in clinical perfectionism, anxiety, depression and eating disorders (Egan and Hine, 2008; Egan et al., 2014; Glover, Brown, Fairburn and Shafran, 2007; Riley, Lee, Cooper, Fairburn and Shafran, 2007; Steele and Wade, 2008; Steele et al., 2013).

A major issue contributing to contention in the literature about clinical perfectionism has been the confusion between a construct and its measurement. The 12-item CPQ (Fairburn, Cooper and Shafran, 2003b) assesses striving to meet standards and effects on self-evaluation when standards are not met. It is brief to complete, has a time frame of one month and the purpose is to measure change in clinical perfectionism with treatment. There is some evidence for the reliability and validity of the CPQ. The internal consistency is good ( = 0.83) in community (Chang and Sanna, 2012) and eating disorder samples (Steele, O’Shea, Murdock and Wade, 2011). The scale has good convergent validity being significantly correlated to PS, CM, SOP, SPP and OOP (Chang and Sanna, 2012; Steele et al., 2011). Dickie, Surgenor, Wilson and McDowall (2012) found a two-factor solution was obtained after items 7 and 8 were excluded, resulting in a 10-item scale. Item 7 was removed as it showed high loadings on both factors, and item 8 was excluded as it had a low item-total correlation where this item correlated positively with half the CPQ items and negatively with the other items. Factor 1 represented striving for standards as it was most strongly related to PS but less so with EC (consisting of CM and DA). Conversely, factor 2 was more strongly related to EC than PS and they concluded this factor assesses concern over not meeting standards and basing self-worth on attainment of standards, which is at the heart of clinical perfectionism. The study by Dickie et al. also found the CPQ had good 4-month test-retest reliability. More recently, Stoeber and Damian (2014) found the CPQ had a two-factor structure capturing perfectionistic strivings and perfectionistic concerns, which we would consider as consistent with the construct of clinical perfectionism as the over-evaluation of striving and attainment of personally demanding standards. An additional study on a large nonclinical sample found that the CPQ captured personal standards, concern over mistakes and the overevaluation of striving (O’Shea, Nehmy, Shafran and Wade, 2014).

The interest in the construct of clinical perfectionism and its measurement warrants further investigation of the CPQ in clinical samples as the construct is based in patient phenomenology. This study aimed to investigate the factor structure, reliability, construct validity, discriminative validity, incremental validity, and readability level of the scale in an eating disorder sample and two separately recruited community samples. In order to demonstrate validity a clear statement is required regarding the proposed strategy (Kane, 2006). We propose construct validity will be demonstrated if clinicians’ ratings of the severity of clinical perfectionism are correlated with the CPQ and if the CPQ correlates significantly with an existing measure of perfectionism. Given perfectionism is associated with psychopathology, we expect that the CPQ will be significantly correlated with eating pathology and negative affect, which will indicate concurrent validity. Discriminative validity will be demonstrated if the CPQ can differentiate between those high and low in negative affect, and with and without eating disorders. Incremental validity is hypothesized with the CPQ predicted to account for variance in negative affect over and above an existing perfectionism measure.

## Study 1: Aims and design

Study 1 included a non-clinical sample and assessed factor structure, reliability, construct validity, discriminative (also referred to as known-groups) validity, incremental validity, and readability. To investigate construct, specifically convergent validity, it was predicted that the CPQ would have a strong positive correlation with the FMPS and the Positive and Negative Affect Schedule (PANAS; Watson, Clark and Tellegen, 1988). To investigate discriminative validity it was proposed that the CPQ would be able to discriminate individuals with high PANAS negative affect scores. Incremental validity was evaluated by investigating whether the CPQ could account for significant variance in the PANAS score over and above the FMPS.

## Method

### Participants

There were 206 participants (74% female, 26% male) aged between 18 and 86 years (*M* = 30.56, *SD* = 13.25) who were recruited online via e-mail and social networking. Participants were a community sample and 29% were university students. Participants predominantly resided in Australia (93%). Participants were a part of an online experimental study investigating standard setting in clinical perfectionism; however, the results of the psychometric properties of the CPQ were not reported in this study (Egan, Dick and Allen, 2012).

### Measures

#### Clinical Perfectionism Questionnaire *(CPQ; Fairburn et al., 2003b)*

This scale has 12 items that assess clinical perfectionism. Items 2 and 8 are reverse-scored. Participants are provided with a definition of perfectionism and asked what areas of their lives this affects other than their eating, weight or appearance. The CPQ items are rated over the past 28 days on a 4-point Likert scale ranging from 1 (not at all) to 4 (all the time). Total scores are the sum of individual items and range from 12 to 48, with higher scores indicating higher clinical perfectionism.

#### Frost Multidimensional Perfectionism Scale *(FMPS; Frost et al., 1990)*

The FMPS consists of 35-items answered on a 5-point Likert scale, ranging from 1 (strongly disagree) to 5 (strongly agree). The FMPS was chosen over other perfectionism measures as the subscales of personal standards and concern over mistakes are close to the definition of clinical perfectionism (Shafran et al., 2002). The original scoring method for personal standards suggested by Frost et al. (1990) was utilized rather than items tapping “pure personal standards” (DiBartolo, Frost, Chang, LaSota and Grills, 2004) in order to enable comparisons across studies where the CPQ has been utilized and which have used the original scoring method for the FMPS rather than pure personal standards. The FMPS is reliable and valid (Frost et al., 1990). Internal consistency in this study was FMPS total = 0.93, PS = 0.85, EC (CM and DA) = 0.92. FMPS total score comprised the Doubts about Actions, Personal Standards, Concern over Mistakes subscale (excluding organization).

#### Positive and Negative Affect Schedule *(PANAS; Watson et al., 1988)*

The PANAS is a 20-item measure of negative and positive affect. Participants rated the extent to which they had experienced each of 20 emotions over the past few weeks on a 5-point scale of “very slightly or not at all” to “very much”. The PANAS is reliable and valid (Watson et al., 1988). The negative affect subscale (PANAS-NA) was used and internal consistency was 0.90.

### Procedure

The study was approved by Human Research Ethics committee at Curtin University. Convenience sampling was utilized through sending emails and posting a link on Facebook to personal contacts and asking their contacts to forward the survey to their own contacts. Personal contacts of the first author who worked in large organizations (e.g. law firms, mining companies) were asked to forward the survey to colleagues. Participants were directed to the online survey page, and given brief instructions followed by the measures.

## Results

Alpha was set at 0.05. The means and standard deviations for the CPQ, FMPS and PANAS-NA can be seen in Table 1. The mean CPQ total was 25.11 (*SD* = 4.77) for females and 25.61 (*SD* = 4.34) for males.

**Table 1.** Means, Standard Deviations, and ranges of the CPQ, FMPS and PANAS

Scale	*M*	*SD*	Sample range
CPQ total	25.25	4.65	15–38
CPQ F1	17.31	3.77	10–30
CPQ F2	7.94	2.05	4–15
FMPS total	95.91	21.42	50–170
PS	22.50	5.89	8–35
EC	30.37	10.65	13–64
CM	20.53	7.83	9–44
DA	9.84	3.77	4–20
PANAS-NA	20.50	7.28	10–44

*Note: SD* = Standard deviation; CPQ = Clinical Perfectionism Questionnaire (Fairburn et al., 2003b); FMPS = Frost et al. Multidimensional Perfectionism Scale (Frost et al., 1990); PS = Personal Standards; EC = Evaluative Concerns; CM = Concern over Mistakes Subscale; DA = Doubts About Actions Subscale; PANAS-NA = Positive and Negative Affect Schedule negative affect subscale (Watson, Clark and Tellegen, 1988)

### Factor structure and internal consistency

An exploratory factor analysis using principal axis factoring (PAF) with promax rotation resulted in two factors accounting for 79% of variance. The two factors were suggested by the reduced correlation matrix eigenvalues rule (Fabrigar and Wegener, 2012) (i.e. two eigenvalues greater than one: 2.50 and 1.37), the scree plot, parallel analysis (O’Connor, 2000), and Velicer's MAP test (O’Connor, 2000), respectively.The correlation between factors was 0.23. The factor structure is shown in Table 2.

**Table 2.** Promax rotated factor structure of the CPQ

		Study 1		Study 2	
No.	Item content (over the past month. . .)	F1	F2	F1	F2
1	Have you pushed yourself really hard to meet your goals?	**.63**	**−.38**	**.81**	−.16
3	Have you been told that your standards are too high?	**.38**	.22	**.41**	.20
6	Have you raised your standards because you thought they were too easy?	**.36**	−.04	**.35**	.11
7	Have you judged yourself on the basis of your ability to achieve high standards?	**.51**	.07	**.40**	**.35**
8	Have you done just enough to get by? (*R*)	**−.37**	**.43**	**−.58**	**.45**
9	Have you repeatedly checked how well you are doing at meeting your standards (for example, by comparing your performance with that of others)?	**.58**	.11	**.38**	**.44**
10	Do you think that other people would have thought of you as a “perfectionist”?	**.54**	.13	**.55**	.01
11	Have you kept trying to meet your standards, even if this has meant that you have missed out on things?	**.63**	−.03	**.64**	.16
2	Have you tended to focus on what you have achieved, rather than on what you have not achieved? (*R*)	.14	**−.36**	.11	**−.43**
4	Have you felt a failure as a person because you have not succeeded at meeting your goals?	.18	.**67**	.06	**.75**
5	Have you been afraid that you might not reach your standards?	.16	.**71**	.26	**.70**
12	Have you avoided any tests of your performance (at meeting your goals) in case you failed?	.12	**.39**	−.07	**.65**

*Notes:* F1 = Factor 1; F2 = Factor 2. Items 2 and 8 are reverse-scored but were not reverse-scored for the factor analysis to aid interpretability. Loadings equal to or greater than |0.3| are bolded

The internal consistency of the CPQ total = 0.71, factor 1 (items loading on factor 1) = 0.71 and factor 2 scale (items loading on factor 2) = 0.63.

### Validity

Pearson's correlations between the measures can be seen in Table 3.

**Table 3.** Pearson correlations between the measures in Study 1

Measures	CPQ total	CPQ Factor 1	CPQ Factor 2
FMPS total	.58***	.43***	.53***
PS	.66***	.64***	.31***
EC	.57***	.35***	.65***
CM	.53***	.32***	.61***
DA	.49***	.31***	.56***
PANAS-NA	.38***	.17*	.54***

*Notes:* **p < .01 ***p < .01 level (two-tailed); CPQ Factor 1 and 2 measures were computed by summing the items that the exploratory factor analysis in Table 3 suggested. CPQ = Clinical Perfectionism Questionnaire (Fairburn et al., 2003b); FMPS = Frost et al. Multidimensional Perfectionism Scale (Frost et al., 1990); PS = Personal Standards subscale of FMPS; EC = Evaluative Concerns (sum of Concern over Mistakes and Doubts about Actions subscales of the FMPS); CM = Concern over Mistakes subscale of FMPS; DA = Doubts about Actions subscale of FMPS PANAS-NA = Positive and Negative Affect Schedule negative affect subscale (Watson, Clark and Tellegen, 1988)

#### Construct validity

The CPQ demonstrated convergent validity as it was strongly correlated with perfectionism and negative affect as seen in Table 3, with large positive correlations with FMPS Total, PS, EC, CM, DA, and PANAS-NA. The partial correlation between PANAS-NA and the factor 1 subscale controlling for the factor 2 subscale was 0.07 (*p* = .34), and 0.53 (*p* < .0001) for factor 2 controlling for factor 1.

#### Discriminative validity

Participants with PANAS-NA scores of ≥26 (75^th^ percentile) were classified “high” and those with scores ≤14 (25^th^ percentile) were classified “low”. An independent samples *t* test showed those with higher PANAS-NA scores (*M* = 9.57, *n* = 49) had significantly higher scores on CPQ factor 2 than those with low PANAS-NA scores (*M* = 6.85, *n* = 47) [*t*(94) = −6.86, *p* < .001] and this effect was large (*d* = 1.39). There was no statistically significant difference between the high (*M* = 17.84, *n* = 49) and low (*M* = 16.48, *n* = 49) groups on CPQ factor 1, *t*(94) = −1.75, *p* = .8, although Cohen's *d* showed a small effect (*d* = 0.36) (Cohen, 1988).

#### Incremental validity

A multiple hierarchical linear regression model showed that the FMPS accounted for 23% of variance in PANAS-NA (*p* < .001), and that the CPQ factors accounted for an additional 11% of variance (*F* change *p* < .001).

### Readability

Flesch readability estimates were obtained with MS Word. The Flesch Reading Ease score was 89.8 and the Flesch-Kincaid Grade Level was 4.1, indicating that the test could easily be read by an average year 4 student (US grade level, student age range 9–10 years).

## Study 2: Aims and design

Study 2 was conducted with an eating disorder sample from a trial of CBT-E (Fairburn et al., 2009) and a community control sample. The aim was to investigate factor structure, construct and discriminative validity. It was proposed construct validity would be demonstrated if there was a significant relationship between clinician ratings of severity of clinical perfectionism and the CPQ, and between the EDE-Q and CPQ. To demonstrate discriminative validity it was predicted that the CPQ would successfully discriminate the patient sample from the controls.

### Participants

There were two groups of female participants: patients with an eating disorder (*n* = 129) and healthy controls (*n* = 80). Clinicians were given a detailed definition of clinical perfectionism and then asked to rate severity using a 4-point Likert scale 4 weeks after the start of treatment (see Fairburn, Cooper, Shafran, Bohn et al., 2008 for further details). The control participants were sent the CPQ and a screening questionnaire to assess the presence of an eating disorder (current or past) or current depression or anxiety.

The patients were engaged in a CBT-E trial and were recruited from consecutive referrals to eating disorder clinics serving Oxfordshire and Leicester, UK. Of the 129 patients, 50 (39%) were from Leicester. Eligibility criteria were having an eating disorder requiring treatment, as judged both by the referring clinician and, subsequently, by the senior eating disorder specialist at each site (ZC, CGF or RLP); being aged 18 to 65 years; having a BMI over 17.5 kg/m^2^. The exclusion criteria were prior evidence-based treatment for an eating disorder, a co-existing Axis 1 disorder that precluded eating disorder-focused treatment, medical instability or pregnancy, and not being available for the duration of treatment.

The mean age of patients was 25.0 years (*SD* = 6.59) and mean BMI was 22.0 (*SD* = 4.34). DSM-IV eating disorder diagnoses were: EDNOS, *n* = 69 (54%), bulimia nervosa, *n* = 53 (41%), binge eating disorder, *n* = 4 (2%), AN, *n* = 3 (1%). The mean age of the control group was 27.7 years (*SD* = 7.14) and mean BMI 23.0 kg/m^2^ (*SD* = 3.29). Controls were significantly older than patients (*t*(197) = 2.71, *p* < .05), but BMI did not differ (*t*(197) = 1.72, *p* > .05). A full description of the patients in this treatment trial is described in Fairburn et al. (2009).

### Procedure

Control participants were recruited from the general population via advertisements placed in Oxfordshire newspapers for females ages 18–45 to complete “some simple questionnaires at home”. This generated 113 enquiries: of those excluded, 6 had a prior/current eating disorder, 11 current anxiety or depression and 5 no longer wished to participate. Questionnaires were sent to 91 women and 80 were returned (response rate of 88%). Patients with an eating disorder completed the CPQ and EDE-Q at the initial assessment sessions prior to engaging in CBT-E.

### Measures

The CPQ was administered and described in Study 1.

#### The Eating Disorder Examination – Questionnaire (EDE-Q; Fairburn and Beglin, 1994)

This contains 28 items that assess eating disorder pathology over the preceding month. There are four subscales: Dietary Restraint, Eating Concern, Shape Concern, and Weight Concern and a global score. It has good reliability and validity (Mond, Hay, Rodgers, Owen and Beumont, 2004).

## Results

Alpha was set at 0.05. Mean CPQ scores can be seen in Table 4.

**Table 4.** Mean and Standard Deviation of CPQ scores in Study 2

Group	*M*	*SD*
CPQ total:		
Patients	28.48	6.19
Controls	24.20	4.45
CPQ F1:		
Patients	17.74	4.63
Controls	16.13	3.37
CPQ F2		
Patients	10.74	2.50
Controls	8.08	2.09

*Note:* CPQ = Clinical Perfectionism Questionnaire (Fairburn et al., 2003b)

### Factor structure and internal consistency

A factor analysis using principal axis factoring with promax rotation revealed a two-factor structure. The two factors were identified with the reduced matrix eigenvalue greater-than-one rule (eigenvalues = 3.64, 1.23), the scree plot, parallel analysis, and Velicer's MAP test, which as in Study 1 all suggested two factors. All items loaded on to the factors with loadings ≥ | 0.3|. The correlation between factors was 0.40.

Internal consistency in the patient sample was 0.82 (total), 0.80 (items loading on factor 1), and 0.64 (items loading on factor 2). In the control sample, internal consistency was 0.73 (total), 0.69 (items loading on factor 1) and 0.72 (items loading on factor 2)

### Validity

#### Construct validity

There was a significant positive correlation between clinicians’ ratings and total CPQ score (*r*s = 0.26, *p* = .005). A one-way ANOVA revealed a significant difference on CPQ total score across patients with different clinical ratings (*F*(3, 108) = 3.22, *p* < .05). Tukey post hoc tests showed no significant differences between the CPQ scores of participants who received severity ratings of “not at all”, “slight” and “moderate”, whereas the CPQ scores of participants who were rated as “major” were significantly higher than those rated as “none”.

#### Discriminative validity

Independent groups *t* tests showed patients’ scored significantly higher than controls on the CPQ total (*t*(199) = 5.74, *p* < .001, mean difference = 4.27), factor 1 (*t*(198) = 2.87, *p* = .004, mean difference = 1.61) and factor 2 (*t*(203) = 7.89, *p* < .001, mean difference = 2.66). Age was included as a covariate in analysis of covariance models because there are findings indicating that perfectionism decreases with age (e.g. Landa and Bybee, 2007). The discriminative validity findings remained the same.

## Discussion

The results of both studies provided evidence of the reliability, construct validity, discriminative validity, incremental validity, and readability of the CPQ. The results indicated that the original 12-item CPQ had acceptable reliability in an eating disorder (α = 0.82) and two community samples (α = 0.71, α = 0.73). Our results are similar to previous studies indicating acceptable consistency (Chang and Sanna, 2012; Steele et al., 2011). Readability was established as age level of 9–10 year olds.

Consistent with previous studies, the CPQ divides into two factors. Factor 1 appears to be assessing predominately the over evaluation of striving as typified by the items “Have you pushed yourself really hard to meet your goals?” and “Have you judged yourself on the basis of your ability to achieve high standards?” This factor correlates significantly with the PS subscale of the FMPS, and also has significant associations with Evaluative Concerns (comprised of the CM and DA subscales from the FMPS). Additional evidence of a complex structure was shown through cross-loadings. Factor 2 appears to be more strongly related to concern over mistakes. The content of these factors are broadly consistent with findings from other studies, notably Dickie et al., 2012, Stoeber and Damian (2014) and O’Shea et al. (2014). It is important to note that these factor solutions support the original description of clinical perfectionism in which over evaluation of striving and reacting to perceived failure with self-criticism are key elements.

There was some evidence for construct validity as the participants who clinicians rated as having “major” clinical perfectionism scored significantly higher scores on the CPQ than those rated as having “none”. The results indicated that there were no differences in CPQ scores between those rated as “slight” and “moderate” on clinical perfectionism, which indicates further work is required to examine the construct validity of the CPQ for those with less severe clinical perfectionism. Furthermore, a limitation is that clinicians ratings were made over a 4-week time period and this may explain the relatively low correlation of *r* = .26 between the ratings.

The CPQ had good construct validity having significant correlations with negative affect and global eating pathology. Discriminative validity was demonstrated with individuals with eating disorders having significantly higher CPQ scores than controls. This was similar to Study 1 where CPQ scores were significantly higher amongst those with higher PANAS-NA scores compared to those with lower PANAS-NA scores. The correlation between perfectionism and the EDE-Q was modest (.36), suggesting that clinical perfectionism and eating disorders are not completely overlapping, which fits with the transdiagnostic theory of eating disorders (Fairburn et al., 2003a) where clinical perfectionism is a core maintaining factor for only a proportion of clients with eating disorders.

The CPQ also had good convergent validity in Study 1 where the CPQ was strongly correlated with the FMPS. These results were similar to Dickie et al. (2012), and Steele et al. (2011), where correlations were also strong. Given that the CPQ has strong correlations with existing measures of perfectionism, this may call in to question the necessity of a new measure. It is argued that the CPQ is necessary as it targets items that directly relate to the clinically relevant aspects of perfectionism, for example, basing self-worth on the attainment of standards. Furthermore, it has the advantage of being sensitive to change in perfectionism over treatment given the short time-frame unlike the multidimensional perfectionism measures. However, clearly further research is required to determine the sensitivity to change that the CPQ demonstrates over treatment.

A strength was that the nonclinical samples were from the community and not only university students. Furthermore, the sample of patients with an eating disorder was relatively large and likely to have been representative. Moreover, the ability of the CPQ to assess clinical perfectionism was validated against a simultaneous but independent rating made by an expert clinician who knew the patient well. Consequently, the test of construct validity is clinically relevant, and not just based on correlations with self-report measures.

There were several limitations. The only patient sample was individuals with an eating disorder where Anorexia Nervosa and underweight Eating Disorder not Otherwise Specified were excluded; thus results are only applicable to those who are not underweight. Information on the CPQ in clinical samples with anxiety and depression is required. Another limitation was community participants were recruited with convenience methods, which can lead to a more homogeneous, biased sample. A further limitation was that sufficient measures that would more comprehensively establish the convergent and divergent validity of the CPQ were not included. It is important in future research to adequately determine the validity of the CPQ by including a wider range of measures. This includes widely used perfectionism measures, as well as additional personality measures such as the 5-factor model to determine, for example, how the CPQ relates to key personality traits of neuroticism and conscientiousness, in addition to a wider range of psychopathology measures than were utilized in the current research. Finally, future research should determine the incremental validity of the CPQ by establishing whether the CPQ accounts for variance in psychopathology above additional existing and widely used measures of perfectionism besides the FMPS.

Overall the results provided evidence for the reliability, validity, and readability of the CPQ. It can be concluded that the CPQ measures what it was intended to: i.e. striving for high standards, concerns over not meeting standards, and basing self-worth on attainment of standards. Future research should examine the CPQ in a large, mixed clinical sample and further determine its utility in capturing change in the treatment of clinical perfectionism.
